# A Randomized, Double-Blind, Biomarker-Selected, Phase II Clinical Trial of Maintenance Poly ADP-Ribose Polymerase Inhibition With Rucaparib Following Chemotherapy for Metastatic Urothelial Carcinoma

**DOI:** 10.1200/JCO.22.00405

**Published:** 2022-08-12

**Authors:** Simon J. Crabb, Syed Hussain, Eileen Soulis, Samantha Hinsley, Laura Dempsey, Avril Trevethan, YeePei Song, Jim Barber, John Frew, Joanna Gale, Guy Faust, Susannah Brock, Ursula McGovern, Omi Parikh, Deborah Enting, Santhanam Sundar, Gihan Ratnayake, Kathryn Lees, Alison J. Birtle, Thomas Powles, Robert J. Jones

**Affiliations:** ^1^ Southampton Experimental Cancer Medicine Centre, University of Southampton, Southampton, United Kingdom; ^2^ University of Sheffield and Sheffield Teaching Hospitals, Sheffield, United Kingdom; ^3^ CRUK Glasgow Clinical Trials Unit, University of Glasgow, Glasgow, United Kingdom; ^4^ The Christie NHS Foundation Trust, Manchester, United Kingdom; ^5^ Velindre Cancer Centre, Cardiff, United Kingdom; ^6^ Northern Centre for Cancer Care, Newcastle upon Tyne, United Kingdom; ^7^ Portsmouth Hospitals NHS Trust, Portsmouth, United Kingdom; ^8^ Leicester Royal Infirmary NHS Trust, Leicester, United Kingdom; ^9^ Dorset Cancer Centre, University Hospitals Dorset NHS Foundation Trust, Poole, United Kingdom; ^10^ University College Hospital, University College London Hospitals NHS Foundation Trust, London, United Kingdom; ^11^ Royal Blackburn Teaching Hospital, East Lancashire Hospitals NHS Trust, Blackburn, United Kingdom; ^12^ Guy's and St Thomas' NHS Foundation Trust, London, United Kingdom; ^13^ Nottingham University Hospitals NHS Trust, Nottingham, United Kingdom; ^14^ Musgrove Park Hospital, Taunton, United Kingdom; ^15^ Maidstone and Tunbridge Wells NHS Trust, Maidstone, United Kingdom; ^16^ Rosemere Cancer Centre, Lancashire Teaching Hospitals NHS Foundation Trust, Preston, United Kingdom; ^17^ St Bartholomew's Hospital, London, United Kingdom

## Abstract

A DNA repair deficiency (DRD) phenotype exists within a subset of metastatic urothelial carcinomas (mUC) predicting benefit from platinum-based chemotherapy. We tested switch maintenance therapy with the poly ADP-ribose polymerase inhibitor rucaparib, following chemotherapy, for DRD biomarker–positive mUC.

**METHODS:**

DRD biomarker–positive mUC patients, within 10 weeks of chemotherapy, and without cancer progression, were randomly assigned (1:1) to maintenance rucaparib 600 mg twice a day orally, or placebo, until disease progression. The primary end point was progression-free survival (PFS). Statistical analysis targeted a hazard ratio of 0.5 with a 20% one-sided α for this signal-seeking trial. PFS (RECIST 1.1) was compared between trial arms, by intention to treat, within a Cox model.

**RESULTS:**

Out of 248 patients, 74 (29.8%) were DRD biomarker–positive and 40 were randomly assigned. A total of 12 (60%) and 20 (100%) PFS events occurred in the rucaparib and placebo arms, respectively (median follow-up was 94.6 weeks in those still alive). Median PFS was 35.3 weeks (80% CI, 11.7 to 35.6) with rucaparib and 15.1 weeks (80% CI, 11.9 to 22.6) with placebo (hazard ratio, 0.53; 80% CI, 0.30 to 0.92; one-sided *P* = .07). In the safety population (n = 39) treatment-related adverse events were mostly low grade. Patients received a median duration of 10 rucaparib or six placebo cycles on treatment. Treatment-related adverse events (all grades) of fatigue (63.2% *v* 30.0%), nausea (36.8% *v* 5.0%), rash (21.1% *v* 0%), and raised alanine aminotransferase (57.9% *v* 10%) were more common with rucaparib.

**CONCLUSION:**

Maintenance rucaparib, following platinum-based chemotherapy, extended PFS in DRD biomarker-selected patients with mUC and was tolerable. Further investigation of poly ADP-ribose polymerase inhibition in selected patients with mUC is warranted.

## BACKGROUND

Platinum-based chemotherapy is a central component for palliative systemic therapy for metastatic urothelial carcinomas (mUC).^1,2^ Although most patients obtain initial clinical benefit, disease progression is inevitable for almost all. Without other intervention, this occurs, on average, within 2-5 months of completing chemotherapy.^2,3^ Subsequent survival and quality-of-life outcomes are typically poor despite second-line intervention.^4,5^ Recent additions to subsequent treatment options, of programmed cell death protein-1–directed immunotherapy, and the nectin- directed antibody drug conjugate enfortumab vedotin have improved survival outcomes.^4,6,7^ However, maintaining clinical benefit from first-line chemotherapy represents an attractive strategy to improve outcomes.^6,8^ Avelumab maintenance immunotherapy, following first-line chemotherapy, improves survival and represents a new standard of care.^6^ However, only a minority of patients appear to benefit from immunotherapy. Therefore, there remains a substantial unmet need for novel, molecularly directed, treatment options for mUC.

**CONTEXT**

**Key Objective**
A minority of patients with metastatic urothelial carcinoma harbor a DNA repair deficiency phenotype, which predicts for benefit from platinum-based chemotherapy. This randomized phase II clinical trial tested whether the poly ADP-ribose polymerase inhibitor rucaparib, as switch maintenance therapy following platinum-based chemotherapy, would improve progression-free survival in patients with DNA repair deficiency biomarker–positive disease.
**Knowledge Generated**
Maintenance rucaparib extended the primary end point of progression-free survival compared with placebo. Treatment was tolerable in this setting.
**Relevance**
Metastatic urothelial carcinoma has a poor prognosis. Development of molecularly targeted treatment options, with predictive biomarkers to aid patient selection, is an important unmet need. To our knowledge, this is the first data to demonstrate efficacy for poly ADP-ribose polymerase inhibition in urothelial carcinoma in the maintenance setting. Broader, prospective validation studies are warranted.

Rucaparib is a small molecule inhibitor of poly ADP-ribose polymerase (PARP) 1, 2 and 3, with activity against *BRCA1* and *BRCA2* altered cancer cell lines. It is approved for relapsed high-grade epithelial ovarian, fallopian tube, or primary peritoneal cancer, either as switch maintenance treatment following platinum-based chemotherapy (restricted to platinum sensitive disease in the European Union), or as a later line of treatment with a *BRCA1* or *BRCA2* alteration.^9‐11^ It is also approved (in the United States) for treatment of metastatic castration resistant prostate cancer with a *BRCA1* or *BRCA2* alteration.^12^

PARP inhibitors, including rucaparib, have been extensively investigated and exhibit clinical activity in various platinum-sensitive cancers.^11,13‐17^ These data support a hypothesis that biomarker selection, either through germline or somatic *BRCA1* or *BRCA2* alteration, or from a wider BRCA-like patient group with a DNA repair deficiency (DRD) phenotype, allows for exploitation of synthetic lethality, and a patient selection strategy.^18,19^ For example, in platinum-responsive ovarian cancer, patients with *BRCA1* or *BRCA2* alteration, or with cancers exhibiting high genome-wide loss of heterozygosity (LOH), experienced improved progression-free survival (PFS) with rucaparib.^11^

PARP inhibition is active against multiple bladder cancer cell lines and xenografts.^20^ Data also support that a mUC subset exhibits a DRD phenotype resulting from defects in various genes including *BRCA1*, *BRCA2*, *ATM*, *RB1*, *PALB2*, *FANCC*, *FANCD2*, and *ERCC2*. A mUC DRD phenotype, for which a clear definition remains to be fully validated, may predict benefit following cisplatin-based chemotherapy in mUC. It also overlaps with potential predictive biomarkers for PARP inhibition.^21‐26^

We hypothesized that PFS would be improved through switch maintenance therapy with rucaparib, for patients who gain clinical benefit following first-line chemotherapy, for mUC that exhibited a DRD biomarker. We tested this in a randomized phase II signal-seeking study within the ATLANTIS clinical trial platform.^8^ The study was performed before the availability of data that now support the use of maintenance avelumab, thereby justifying a placebo control arm.^6^

## METHODS

### Study Design and Participants

This study was a randomized comparison within the ATLANTIS trial, which has been described previously.^8^ In brief, ATLANTIS is an adaptive, multicomparison, clinical trial platform. It tests multiple, biomarker-selected switch maintenance therapies for patients with mUC, and without disease progression after completing four to eight platinum-based chemotherapy cycles, in a series of parallel, randomized, double-blind, phase II comparisons. A prescreening phase, from any point after diagnosis of mUC, allows for testing of archival tumor samples for multiple biomarkers for allocation to respective randomized comparisons. Patients found to have a positive DRD biomarker were eligible for subsequent screening to enter the rucaparib randomized comparison, which is described here. Other ATLANTIS randomized comparisons will be reported elsewhere.

For the rucaparib randomized comparison, the composite DRD biomarker was positive with any of the following present: ≥ 10% genome-wide LOH^27^ and/or alteration in a defined DRD associated gene (*ATM*, *BARD1*, *BRCA1*, *BRCA2*, *BRIP1*, *CDK12*, *CHEK2*, *FANCA*, *NBN*, *PALB2*, *RAD51*, *RAD51B*, *RAD51C*, *RAD51D*, and *RAD54L*) within an archival tumor sample and/or prior confirmation of a germline alteration in *BRCA1* or *BRCA2* (germline testing was not performed for trial entry purposes). Biomarker testing used the FoundationOne next-generation sequencing assay.^28,29^

Other eligibility requirements included stage IV (stage T4b and/or N1-3 and/or M1), histologically confirmed urothelial carcinoma unsuitable for curative treatment options. Patients were age 16 years or older, with Eastern Cooperative Oncology Group performance status 0-2, and were required to commence study treatment within three to 10 weeks of completing four to eight cycles of first-line platinum-containing chemotherapy given with palliative intent. Prior neoadjuvant or adjuvant treatments were not counted as first-line chemotherapy. Prior immunotherapy was permitted. Adequate organ function required neutrophils ≥ 1.5 × 10^9^/L, platelets ≥ 100 × 10^9^/L, hemoglobin ≥ 9 g/dL, bilirubin ≤ 1.5 times the institutional upper limit of normal (ULN), AST and ALTs ≤ 3 × ULN (≤ 5 × ULN with liver metastases), serum albumin ≥ 28 g/L, and creatinine clearance ≥ 30 mL/min. Patients were excluded for cancer progression during, or at completion of, first-line chemotherapy, prior treatment with a PARP inhibitor, pre-existing gastrointestinal disorders or conditions that would affect oral administration, or absorption, of rucaparib, previous myelodysplastic syndrome, symptomatic or untreated central nervous system metastases, or use of oral anticoagulants or platelet inhibitors. Complete eligibility criteria are listed in the Protocol (online only).^30^

The study was undertaken in accordance with the Declaration of Helsinki and Good Clinical Practice guidelines, and approved by West of Scotland Research Ethics Committee (16/WS/0197). All patients provided written informed consent.

### Random Assignment and Masking

Patients were randomly assigned (1:1), on a double-blind basis, to treatment with rucaparib 600 mg twice a day orally, or matched placebo, to commence within 10 weeks of first-line chemotherapy. Random assignment was stratified via minimization factors (cisplatin-based *v* non–cisplatin-based first-line chemotherapy; Eastern Cooperative Oncology Group performance status 0 *v* 1 *v* 2; complete or partial response to first-line chemotherapy *v* stable disease; presence of visceral metastases; presence of measurable disease; and investigational site).

### Procedures

Treatment continued until disease progression as assessed by local investigators by RECIST version 1.1, need for new anticancer systemic therapy, unacceptable toxicity, or withdrawal of consent. Dose modifications and delays were permitted for hematologic and nonhematologic toxicities as detailed within the protocol.^30^ Disease evaluation was via cross-sectional imaging of the chest, abdomen, and pelvis at baseline, then every 12 weeks in year 1, every 16 weeks in year 2, and then every 24 weeks until disease progression. Patients were reviewed every 4 weeks until disease progression and then for survival status only.

### Outcomes

The primary end point was investigator-assessed PFS measured as the time from random assignment until progressive disease as defined by RECIST version 1.1 or death from any cause. Secondary end points included overall survival (time from random assignment until death from any cause), confirmed response rates (RECIST version 1.1), maximum percentage decrease in measurable disease, safety, and tolerability (Common Terminology Criteria for Adverse Events, version 4.03).

### Statistical Analysis

The rucaparib comparison statistical design assumed a median PFS in placebo-allocated patients of 5.4 months (adjusted for prognostic impact of biomarker selection).^3^ We targeted a hazard ratio (HR) of 0.5 on the basis of effect size seen with PARP inhibitors in a similar setting in ovarian cancer using a similar DRD biomarker signature for patient selection.^11^ This required ≥ 39 PFS events by recruiting 48 patients over 27 months with 8 months of subsequent follow-up. This provided 90% power, at a 20% one-sided level of statistical significance (or equivalently 80% power at the 10% level of statistical significance).^31^ As a result of a recruitment hiatus in UK centers from March 2020 because of the global pandemic, and emergent data to support use of avelumab immunotherapy in the maintenance setting,^6^ recruitment was discontinued after random assignment of 40 patients. Our statistical parameters were therefore prospectively revised for a single and final analysis to occur after reaching ≥ 30 PFS events. This reduced the power to 85.4%. All other parameters remained unchanged. Analysis was conducted on an intention-to-treat (ITT) basis for all efficacy end points with PFS compared between the treatment allocation arms within a Cox model incorporating baseline minimization factors. HRs are presented adjusted for minimization factors (except investigational site as only one site contributed more than five patients). Worst toxicity grades experienced during treatment were compared using the Mann-Whitney *U* test. The study had oversight from an independent data monitoring committee. A nonbinding test for futility was reviewed by the independent data monitoring committee after half of the PFS events had occurred on the basis of a Lan-DeMets monitoring boundary with an O'Brien-Fleming stopping rule.

ATLANTIS is registered with the ISRCTN registry (ISRCTN25859465).

## RESULTS

**FIG 1.**
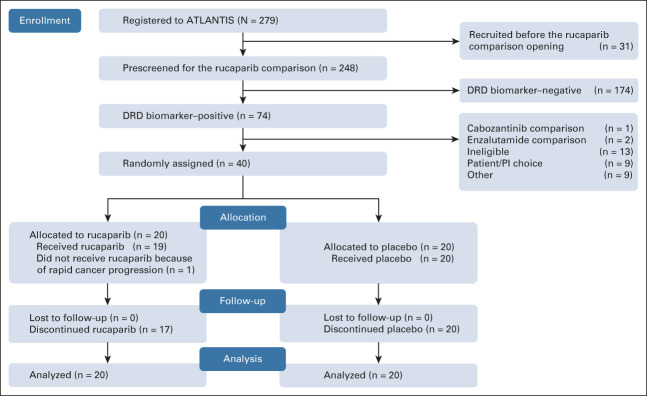
CONSORT diagram. DRD, DNA repair deficiency; PI, principal investigator.

**FIG 2.**
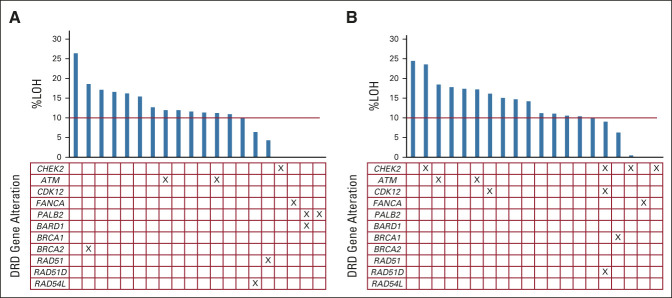
DRD biomarker status for individual patients (columns) for %LOH and designated somatic genes in patients allocated to (A) rucaparib or (B) placebo. %LOH, percent genome-wide loss of heterozygosity; DRD, DNA repair deficiency.

Prescreening for the rucaparib randomized comparison occurred between November 24, 2017, and February 2, 2021. Data cutoff for this analysis occurred on November 17, 2021. A total of 248 patients underwent biomarker prescreening, of whom 74 (29.8%) were DRD biomarker–positive. Forty of these were randomly assigned within the rucaparib comparison (Fig 1). Of patients randomly assigned, the DRD biomarker was positive because of ≥ 10% LOH in 22 (55%), a somatic gene alteration in 11 (27.5%), or both in seven (17.5%). Individual biomarker status for randomly assigned patients is shown in Figure 2 (no patients were known to have a germline *BRCA1* or *BRCA2* alteration). At data cutoff, three patients (15%) were continuing to receive rucaparib, with none remaining on placebo. Median duration of follow-up for patients who remained alive was 94.6 weeks (range, 11.4-148.9 weeks).

Within the ITT population, median age was 70.5 years (range, 53-86 years) with 35 (87.5%) having a primary bladder cancer and 18 (45%) visceral metastases. Twenty-five patients (62.5%) had received cisplatin-based chemotherapy and 36 (90%) had achieved an objective response to first-line chemotherapy. Patient characteristics are presented in Table 1 and were reasonably balanced between allocated treatment arms.

**TABLE 1.**
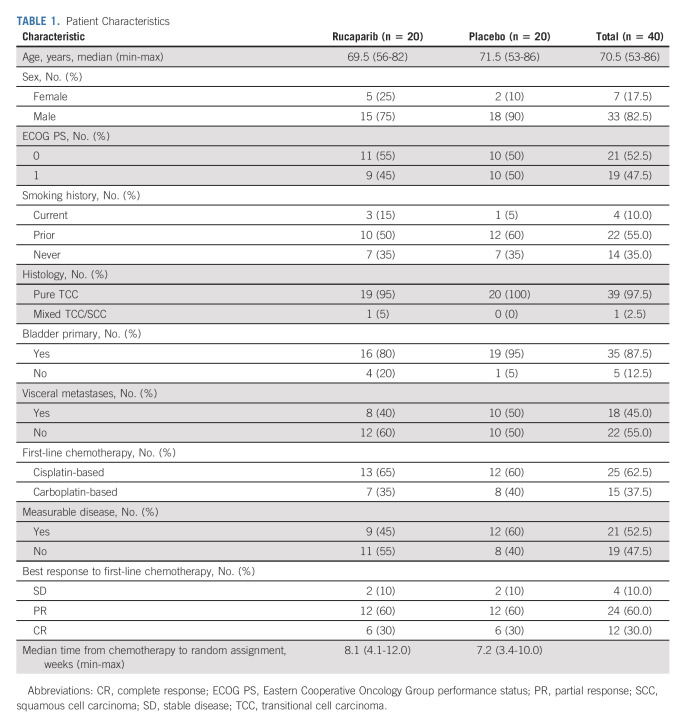
Patient Characteristics

**FIG 3.**
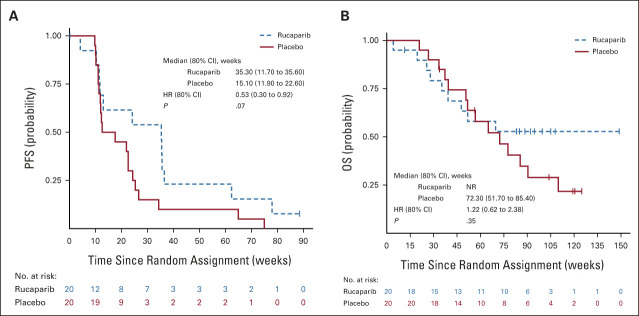
Kaplan-Meier curves of (A) progression-free survival and (B) overall survival. HR, hazard ratio; NR, not reached.

**FIG 4.**
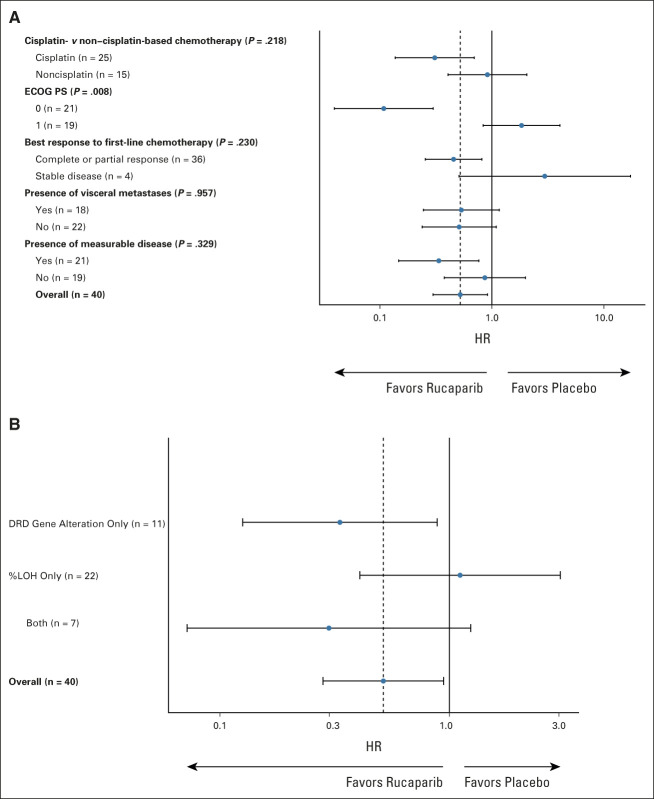
Forest plot to show variation in progression-free survival treatment effect with respect to (A) minimization factors and (B) whether patients were found to be DRD biomarker–positive on the basis of an alteration in a prespecified list of DNA repair genes, or because of ≥ 10% LOH, or both. %LOH, percentage genome-wide loss of heterozygosity; DRD, DNA repair deficiency; ECOG PS, Eastern Cooperative Oncology Group performance status; HR, hazard ratio.

Twelve (60%) patients receiving rucaparib and 20 (100%) receiving placebo have had PFS events. Median PFS was 35.3 weeks (80% CI, 11.7 to 35.6) with rucaparib and 15.1 weeks (80% CI, 11.9 to 22.6) with placebo with an adjusted HR of 0.53 (80% CI, 0.30 to 0.92; one-sided *P* = .07; unadjusted HR, 0.51 [0.31 to 0.83]; one-sided *P* = .04; Fig 3A). A forest plot illustrating how treatment effect varied with respect to factors in the minimization algorithm is shown in Figure 4A.

Nine (45%) and 14 (70%) patients had died in the rucaparib and placebo arms, respectively. Median overall survival was not reached in the rucaparib treatment arm and 72.3 weeks (80% CI [51.7 to 85.4] for placebo with an adjusted HR of 1.22 [80% CI, 0.62 to 2.38]; *P* = .35; unadjusted HR, 0.70 [80% CI, 0.4 to 1.2]; *P* = .21; Fig 3B).

**FIG 5.**
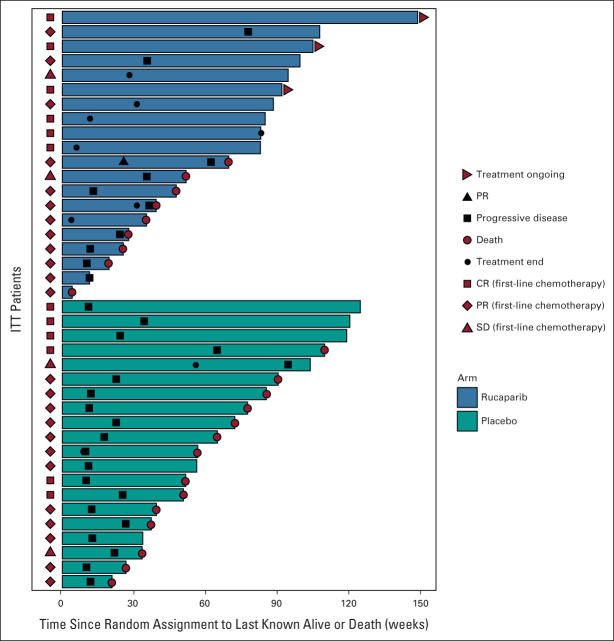
Swimmers plot to indicate time on treatment, treatment response, and time to death. CR, complete response; ITT, intention-to-treat; PR, partial response; SD, stable disease.

A total of 36 (90%) patients in the ITT population had already achieved an objective radiologic response to first-line chemotherapy with 12 (60%) partial responses and six (30%) complete responses in both treatment groups. A single further confirmed partial response occurred in one patient (5%) treated with rucaparib. No other objective radiologic responses to treatment occurred on study, in either treatment group. A swimmer's plot depicting time on treatment, response outcomes, and time to death is shown in Figure 5. Maximal percentage reduction in measurable disease was similar between treatment arms with medians of −5.8% (interquartile range [IQR], −21.2-36.3) and −4.9% (IQR, −17.9-37.7) for rucaparib and placebo groups, respectively.

As an exploratory post hoc analysis, we assessed PFS within subgroups defined by the components of the DRD biomarker. Subgroup sizes limit definitive conclusions, but a trend is seen toward benefit with use of rucaparib in those patients with a DRD gene alteration (irrespective of LOH status) but not with high percentage LOH alone (Fig 4B, Appendix Table A1, online only).

The safety population comprised 39 patients (one patient allocated to rucaparib suffered cancer progression before commencing treatment). Patients received a median duration of 10 cycles (28 days) of treatment with rucaparib versus six with placebo. The median percentage of maximum intended dose (reflecting dose reduction and omission *v* 600 mg twice a day for all prescribed cycles) was 87.3% (IQR, 75.0-98.2) for rucaparib and 91.3% (IQR, 82.0-97.9) for placebo. Three patients in the rucaparib arm continue study treatment and none in the placebo group. Five (26.3%) on rucaparib and one (5%) on placebo have discontinued treatment because of treatment-related toxicity or patient choice. At least one dose reduction was required in seven (36.8%) patients receiving rucaparib and four (20%) receiving placebo.

Treatment-related adverse events were mostly low grade (Table 2). Considering all grades, fatigue (63.2% *v* 30.0%, *P* = .03), nausea (36.9% *v* 5.0%, *P* = .03), rash (21.1% *v* 0%, *P* = .04), and raised alanine aminotransferase (57.9% *v* 10%, *P* = .003) were more common with rucaparib than with placebo. There were no treatment-related deaths.

**TABLE 2.**
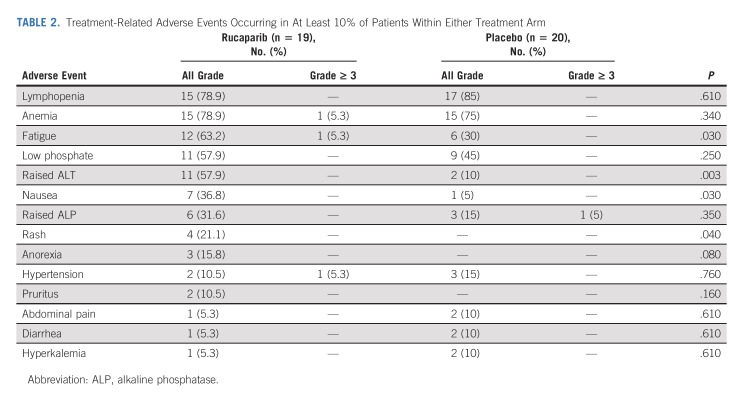
Treatment-Related Adverse Events Occurring in At Least 10% of Patients Within Either Treatment Arm

Second-line treatment for cancer progression, following trial participation, for patients in the rucaparib group was with programmed cell death protein-1–directed checkpoint inhibitor immunotherapy in three (25%) patients and paclitaxel in one (8%) of the 12 who had experienced a PFS event. For the placebo group, 11 of 20 (55%) patients received immunotherapy (with one of these also receiving erdafitinib and an experimental agent).

## DISCUSSION

In this efficacy signal-seeking phase II trial, we found that switch maintenance treatment with rucaparib resulted in clinically meaningful improvement in the primary end point of PFS, despite small numbers. The added benefit with PARP inhibition appears to have been primarily through maintenance of established clinical benefit from prior chemotherapy, rather than through further reductions in cancer volume and objective radiologic response. This mirrors experience with maintenance immunotherapy for mUC, where objective response rates were also low, and is relevant to clinical trial design in the maintenance setting.^6^ This pattern of anticancer effect was to be anticipated within a population selected not only through clinical benefit from chemotherapy, but also for a biomarker for a DRD phenotype. Indeed, we saw a 90% objective response rate to prior chemotherapy, for example, compared with 72% in the JAVELIN Bladder 100 maintenance avelumab trial (where biomarker selection was not employed).^6^ Impact of biomarker selection was also reflected in our control arm median PFS of 15.1 weeks versus 2.1 months in JAVELIN Bladder 100. The additional toxicity associated with treatment was consistent with prior experience for PARP inhibition, and rucaparib specifically. Rucaparib appears to have an acceptable safety and tolerability profile for administration in this clinical setting.

Rucaparib monotherapy has been tested in mUC previously in the single-arm, phase II, ATLAS trial, which enrolled patients in the second- or third-line palliative setting. Patients were not selected by biomarker status or prior clinical benefit from platinum chemotherapy. The trial did not meet its primary end points, with no confirmed objective responses in the unselected ITT population, or within a subset exhibiting ≥ 10% genome-wide LOH (from tumor samples taken at trial entry).^27^ This LOH cut point was selected for ATLAS, and was subsequently included within our composite biomarker, on the basis of data that it optimally differentiated survival benefit for urothelial carcinoma following platinum-based chemotherapy.^27^ In addition, in the BISCAY phase Ib platform study, for second or subsequent line treatment of mUC, one of six biomarker selected treatment arms included nonrandomized cohorts receiving the PARP inhibitor olaparib combined with durvalumab immunotherapy. Outcomes did not appear qualitatively different, either compared with durvalumab alone, or with respect to presence or absence of a 15-gene panel biomarker for DNA homologous recombination repair deficiency, although response rates appeared higher in the DRD population (35% *v* 9%).^32^ The discordance of results between these studies and ours may potentially relate to our use of a first-line switch maintenance treatment strategy. This results in a cohort with established, and current, chemotherapy benefit rather than the need for salvage at the point of cancer progression in a mixed population of patients, which includes innate or acquired platinum resistance.

Since ATLANTIS was launched, therapeutic options in this setting have evolved with an established survival advantage for maintenance avelumab immunotherapy in unselected patients.^6^ Further development of PARP inhibition may therefore need to be within the context of immunotherapy combinations. This practical reality may have implications for both the functionality, and potentially even the need, for patient selection biomarkers. It may also affect aspects of tolerability, for a patient population with relative frailty, who have recently completed chemotherapy.

Our study has some limitations. Our planned sample size was affected through the global pandemic and emergent new data for avelumab immunotherapy for this clinical setting. Our statistical analysis was adjusted prospectively to allow for meaningful data to be presented. Nevertheless, the sample size was small, and the possibility of type 1 error is therefore relatively high, and we had some imbalances in prior cisplatin exposure, performance status, and presence of visceral and measurable disease. At the point that we designed this study, there was no standard, and no prior clinical data, to guide the design for a biomarker selection approach. As such, the gene panel and utilization of a LOH cut point were based on the known genomics of mUC and extrapolation from data in other cancers. Future development of PARP inhibition for mUC should ideally look to validate, and evolve, our understanding of the optimal approach and need for a predictive biomarker for patient benefit. Exploratory subgroup analysis, which should be interpreted with caution, suggests that the benefit derived from rucaparib may have been limited to within those with a defined gene alteration. We suggest that a gene panel approach alone, without incorporating LOH, may be optimal for patient selection. Future prospective trials will be required for this to be validated as a predictive biomarker. This trial also lacked central radiology review, although this was partially mitigated by the double-blind design.

In conclusion, PARP inhibition with rucaparib extended PFS as a switch maintenance approach in biomarker selected patients with mUC, despite small numbers in the trial. Further development of PARP inhibition in mUC is now warranted. This may require combination strategies and will require further scrutiny of patient genomic phenotypes to optimally integrate into the increasingly complex treatment pathway for this disease.

## Figures and Tables

**TABLE A1.**
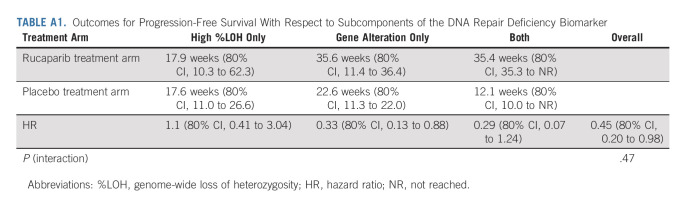
Outcomes for Progression-Free Survival With Respect to Subcomponents of the DNA Repair Deficiency Biomarker

## Data Availability

The ATLANTIS investigators are committed to sharing data with others in the field, who wish use it for high-quality peer-reviewed research. We are happy to consider proposals from researchers and will share deidentified individual-patient data to the maximum extent, subject to individual study constraints relating to (1) ethical approval and informed consent; (2) contractual and legal obligations, including a data sharing agreement; and (3) publication timelines (data will not normally be shared before publication of the primary results). All proposals will be reviewed for their scientific merit by the trial management group. Only data relevant to the objectives of a particular proposal will be provided. To make a proposal, please contact CRUK Glasgow CTU via e-mail at mvls-ctu-enquiries@glasgow.ac.uk.
